# Comparisons of School-Day Glycemia in Different Settings for Children with Type 1 Diabetes Using Continuous Glucose Monitoring

**DOI:** 10.1155/2023/8176606

**Published:** 2023-03-09

**Authors:** Christine A. March, Michelle Nanni, James Lutz, Madison Kavanaugh, Kwonho Jeong, Linda M. Siminerio, Scott Rothenberger, Elizabeth Miller, Ingrid M. Libman

**Affiliations:** ^1^Department of Pediatrics, University of Pittsburgh, Pittsburgh, PA, USA; ^2^School of Medicine, University of Pittsburgh, Pittsburgh, PA, USA; ^3^School of Arts and Sciences, University of Pittsburgh, Pittsburgh, PA, USA; ^4^Department of Pediatrics, UPMC Children's Hospital of Pittsburgh, Pittsburgh, PA, USA; ^5^Center for Research on Health Care Data Center, University of Pittsburgh, Pittsburgh, PA, USA; ^6^Department of Medicine, University of Pittsburgh, Pittsburgh, PA, USA

## Abstract

**Objective:**

Using continuous glucose monitoring (CGM), we examined patterns in glycemia during school hours for children with type 1 diabetes, exploring differences between school and nonschool time.

**Methods:**

We conducted a retrospective analysis of CGM metrics in children 7–12 years (*n* = 217, diabetes duration 3.5 ± 2.5 years, hemoglobin A1c 7.5 ± 0.8%). Metrics were obtained for weekday school hours (8 AM to 3 PM) during four weeks in fall 2019. Two comparison settings included weekend (fall 2019) and weekday (spring 2020) data when children had transitioned to virtual school due to COVID-19. We used multilevel mixed models to examine factors associated with time in range (TIR) and compare glycemia between in-school, weekends, and virtual school.

**Results:**

Though CGM metrics were clinically similar across settings, TIR was statistically higher, and time above range (TAR), mean glucose, and standard deviation (SD) were lower, for weekends and virtual school (*p* < 0.001). Hour and setting exhibited a significant interaction for several metrics (*p* < 0.001). TIR in-school improved from a mean of 40.9% at the start of the school day to 58.0% later in school, with a corresponding decrease in TAR. TIR decreased on weekends (60.8 to 50.7%) and virtual school (62.2 to 47.8%) during the same interval. Mean glucose exhibited a similar pattern, though there was little change in SD. Younger age (*p* = 0.006), lower hemoglobin A1c (*p* < 0.001), and insulin pump use (*p* = 0.02) were associated with higher TIR in-school.

**Conclusion:**

Although TIR was higher for weekends and virtual school, glycemic metrics improve while in-school, possibly related to beneficial school day routines. Keywords: type 1 diabetes, school health, continuous glucose monitoring, time in range, glycemic control.

## 1. Introduction

Schools provide a supportive and consistent environment for children with type 1 diabetes in numerous ways. Like all youth, children with diabetes spend nearly half their weekday waking hours in school, where they have established routines for mealtimes and classroom activities. In school, children with diabetes are largely under the care of school nurses or other trained staff who can assist them with both routine and urgent diabetes care tasks [1]. As schools support type 1 diabetes management, some centers have piloted interventions in the school setting, including school nurse administration of long-acting insulin, diabetes education in school, telemedicine, and care coordination [2–6]. Though many have demonstrated some improvements in patient- or nurse-reported measures, the impact on hemoglobin A1c in the short term has been inconclusive [7].

One challenge is that hemoglobin A1c is insufficiently dynamic to assess changes in school-day glycemia resulting from interventions targeted to that setting. A more useful assessment may be through modern continuous glucose monitoring (CGM), increasingly used in pediatric diabetes care [8]. In addition to providing time spent in certain glucose ranges, CGM generates other metrics which are indicators of glycemic variability, including standard deviation (SD) and coefficient of variation for glucose (CV), among others. These metrics, particularly low time in range (TIR) and high SD, may be associated with increased risk of micro- and macrovascular complications in cross-sectional analyses [9]. CGM metrics have quickly become important adjunct markers to hemoglobin A1c for type 1 diabetes. The current recommendation from international consensus guidelines for children, similar to adults, is that 70% of readings, or greater than 16 hours/day, be in range (glucoses 70–180 mg/dL) [10].

CGM metrics can be subdivided and evaluated at different time periods throughout the day, helping users understand how blood glucoses vary in relation to activities and food intake. With the ability to obtain hourly measures of glycemia, CGM makes possible more granular insight into glycemic variations within the specific window of the school day for use in clinical care or research, which, to our knowledge, has never been studied. An improved understanding of school-day glycemia and related factors may help to target, implement, and evaluate the short- and long-term implications of interventions for school-based diabetes management in the future.

The objectives of this study were to examine glycemia in children with type 1 diabetes during school hours using CGM metrics, investigate predictors of TIR in school, and compare glycemic control in school to nonschool time. We hypothesized that (1) CGM-determined percent TIR during school hours would be suboptimal (<70%) in children, (2) clinical predictors, such as lower hemoglobin A1c and insulin pump use, would be associated with higher percent TIR, and (3) CGM metrics would suggest better glycemic control in-school compared to nonschool time.

## 2. Methods

### 2.1. Study Design

This is a retrospective study of CGM metrics recorded during school hours for children with type 1 diabetes who receive care in our academic diabetes center. Our center provides care for approximately 2200 children and adolescents with type 1 diabetes, with 250–300 children newly diagnosed each year. Based upon a recent review of our data, approximately 68% of this population uses a CGM. Our center relies upon published, international consensus guidelines for recommended targets of hemoglobin A1c and CGM metrics in our approach to management [10, 11].

Data were collected from an existing clinical tool used to access CGM data, the secure cloud system, Dexcom CLARITY (v3.25.1, Dexcom Inc, US). Hourly CGM reports were obtained to isolate data between 8 AM and 3 PM (labeled as “school hours”) based on local school start and end times. For each subject, separate CGM reports were generated from CLARITY to represent glycemia while in-school and out of school.

To assess glycemia in school, CGM data were collected for weekdays only (Monday–Friday) during a four-week period in fall 2019 (9/30/2019–10/27/2019). This date range was chosen as it falls between the beginning of the school year and peak influenza season and avoids many holidays, trying to make it as representative as possible for the general school year. Glycemic metrics during this time are reported as “in-school.”

CGM data were collected at two nonschool times with the same hours (between 8 AM to 3 PM) for comparison purposes. First, we collected weekend (Saturday-Sunday) data during the same four-week period in fall 2019. Second, we collected weekday (Monday–Friday) data during a four-week period during the spring 2020 (4/6/2020–5/1/2020), when schools were largely closed due to the pandemic, and children were doing virtual learning at home. Glycemic metrics from these times are reported as “weekend” and “virtual school,” respectively. This study was deemed exempt by the University of Pittsburgh Institutional Review Board (PRO#20010213).

### 2.2. Study Subjects

Children with a diagnosis of type 1 diabetes between 7 and 12 years of age who use a Dexcom G5 or G6 were included in this study. This age range reflects an elementary and middle school-aged population who are more likely to require assistance from trained school staff for their diabetes management. Younger children were excluded as they may participate in half-day programs, and older children were excluded as they are more often permitted to be independent in their care.

We first queried our Dexcom CLARITY database for children who were between 7 and 12 years old as of 9/30/2019 with a diagnosis of type 1 diabetes (ICD-10 code E10). The initial query identified 465 possible subjects; preliminary exclusions were for duplicate records (*n* = 6), incorrect diagnosis (*n* = 10), diabetes diagnosis/CGM initiation after 9/30/2019 (*n* = 60), home/cyber school (*n* = 18), not followed by our clinic (*n* = 7), data sharing turned off (*n* = 47), and missing >1 week of data indicating a lapse in CGM wear (*n* = 84), leaving a sample of 233 meeting inclusion criteria. For analyses, children were excluded if they had less than 70% wear time during school hours across the 4-week period or had no clinical encounter at our center within 6 months of the CGM data range.

### 2.3. Study Measures and Outcome

Dexcom CGM provided overall statistics as well as hourly metrics (12 readings per hour per day), which were aggregated over school hours. Primary analyses assessed TIR (70–180 mg/dL), time below range (TBR, level 1 : 54–69 mg/dL; level 2: <54 mg/dL), time above range (TAR, level 1 : 181–250 mg/dL; level 2: >250 mg/dL), mean glucose, glucose SD, and CV.

Contemporaneous data were extracted from the electronic health record (EHR) by two reviewers to identify demographic and health-related information for each participant. Background characteristics included age, race, sex, insurance status (private vs. public), and ZIP code. ZIP code was used to obtain median household income from US census data and area deprivation index (ADI) national percentile as measures of socioeconomic status [12, 13]. Health-related variables included duration of diabetes, hemoglobin A1c, insulin regimen (multiple daily injections or insulin pump), insulin dose, and anthropometric measurements (e.g., body mass index z-score). We did not distinguish among insulin pump types, as no subjects were using an automated insulin delivery system (the first to pair with Dexcom, the Tandem X2 pump™ with *Control IQ*™ algorithm, was not approved for children under the age of 14 years until the summer of 2020).

EHR data were collected from the diabetes visit immediately preceding and immediately following the start date of in-school CGM data extraction (9/30/2019) to identify any clinically significant changes (e.g., insulin pump start) and detect large swings in numeric data (e.g., insulin dose per kilogram). Hemoglobin A1c and insulin dose per kilogram were averaged over the two clinic visits for analyses. The number of diabetes visits in the year preceding the start date of CGM data extraction was also collected. A senior investigator was available to adjudicate any issues in data extraction.

A second chart review for spring 2020 data was conducted to identify any changes in the insulin regimen. As clinical encounters with our diabetes center were mostly virtual at that time, we were unable to obtain updated anthropometric measures or hemoglobin A1c values.

### 2.4. Data Analysis

Descriptive statistics were used to summarize background characteristics and the CGM metrics for in-school, weekends, and virtual school. We reported the metrics for the school day (8 AM to 3 PM) and hourly to identify any patterns; 24-hour data were also available and compared across settings (Supplement 1). Hourly trends in TIR, TBR, and TAR were compared by age (< or ≥10 years), hemoglobin A1c (≤SD or >7%), and insulin regimen (multiple daily injections or insulin pump). As the number of readings varied by day and hour of the day within each child, hourly metrics of glucose (mean, SD, and CV) were computed in a weighted fashion with weights proportional to the number of readings available to ensure that times with fewer readings were not overrepresented.

We examined relationships between sociodemographic/clinical factors and in-school TIR while adjusting for time trends using multilevel linear modeling. First, we constructed a “base” model with in-school TIR as the outcome, fixed effects representing a polynomial in time (hour), a random intercept for the subject, and a random coefficient for time (hour). Using an unstructured covariance pattern, the model was fitted via maximum likelihood estimation. Likelihood ratio tests were performed to determine the optimal number of polynomial terms, revealing that a quadratic polynomial in time fits significantly better than linear time (*p* < 0.001), but the inclusion of a cubic time effect was not significant (*p*=0.1314). Thus, our base model included fixed effects for hour and hour-squared.

We then investigated the impact of sociodemographic/clinical characteristics on in-school TIR by augmenting the base model with predictors in three steps: (1) “unadjusted” models examining the univariate relationship between each predictor and TIR, while always accounting for time effects; (2) a fully adjusted model including all predictors and time effects; and (3) a final, parsimonious model including all time effects and predictors found to be significant at the *p* < 0.20 level in the fully adjusted model. A relatively liberal criterion for inclusion in the final model (*p* < 0.20) was chosen to avoid omitting potential confounders.

To assess for differences in CGM metrics (TIR, TAR, TBR, mean glucose, SD, CV) between settings during school hours, similar modeling procedures as those above were employed. We constructed base models which included fixed effects for an hour, hour-squared, and an indicator variable for setting (in-school vs. weekend or in-school vs. virtual school). We also included an interaction term between hour and setting, as the rate of change in outcome (i.e., slope) may vary between settings. In other words, the inclusion of the interaction allows us to infer whether temporal trajectories differ significantly between in-school and weekend hours. We then included sociodemographic/clinical characteristics in a fully adjusted model. Levels 1 and 2 for TBR and TAR were condensed for analyses. Observed means and standard deviations are reported with corresponding *p*-values from regression models.

Median household income and ADI national percentile were found to be highly correlated (Spearman's rho = −0.727); therefore, ADI was selected for all modeling procedures. Analyses were completed in Stata/SE 17.0. We assumed a significance level of 0.05, and no adjustments were made for multiplicity. Data were analyzed from September–December 2021.

## 3. Results

A total of 233 subjects met the inclusion criteria. One subject was excluded due to no clinical encounter within six months of the fall 2019 CGM extraction period. Fifteen subjects were excluded due to <70% CGM wear on in-school weekdays, leaving an in-school cohort of 217. For the two comparison groups, 8 subjects were excluded due to <70% CGM wear during weekends and virtual school, leading to final cohorts of 209 for each group. The excluded subjects were similar to those included in terms of race, sex, age, and hemoglobin A1c. Background characteristics are summarized in Table 1. CGM metrics for in-school, weekend, and virtual school between 8 AM and 3 PM are presented in Tables 2–4, respectively. The 24-hour metrics for each setting are included in Supplement 1. For 24-hour data comparisons, the only significant differences between settings included a slightly lower SD (weekends and virtual school) and slightly higher TBR (weekends only) compared to in-school.

### 3.1. In-School CGM Metrics

During school hours, the mean TIR for the sample was 51.0 ± 23.4%. Only 10 (5%) of the subjects met a TIR goal of ≥70% in school for school hours. Mean TBR was 1.3 ± 2.5%, meeting the recommended threshold of <4%. TAR during school hours was 47.6 ± 24.2%, and approximately 40% of that time was spent with a blood glucose >250 mg/dL (level 2).

For the hourly data, there was a notable increase in TIR from 40.9 ± 23.6% to a peak of 58.0 ± 22.7% and decrease in TAR from 58.2 ± 24.3 to a nadir of 40.5 ± 23.5% over the first four hours of school, at which time these metrics became steadier. Levels 1 and 2 TAR both decreased by approximately 10 percentage points during in-school hours. These findings were mirrored in the mean glucose results, though the standard deviation and coefficient of variation did not change significantly over the course of the day. This pattern did not differ by age, hemoglobin A1c, or pump use, though those with a hemoglobin A1c in target overall had higher TIR.

In univariate regression models, time (hour of the day and hour-squared, both (*p* < 0.001), younger age (*p*=0.001), shorter duration of diabetes (*p* < 0.001), lower insulin dose per kilogram (*p* < 0.001), and lower hemoglobin A1c (*p* < 0.001) were associated with higher TIR (Table 5). All but insulin dose and duration of diabetes remained significant in the parsimonious model. Insulin pump use was not significant in univariate analysis (*p*=0.70) but became significant in the fully adjusted and parsimonious models (*p*=0.02) after adjusting for other variables. In univariate analyses, race (*p*=0.24), body mass index z-score (*p*=0.83), insurance status (*p*=0.77), and ADI national percentile (*p*=0.38) were not significant predictors of TIR. These predictors were also not significant in adjusted and parsimonious models (all *p* > 0.34).

### 3.2. Comparison of Weekend CGM Metrics

**Figure 1:**
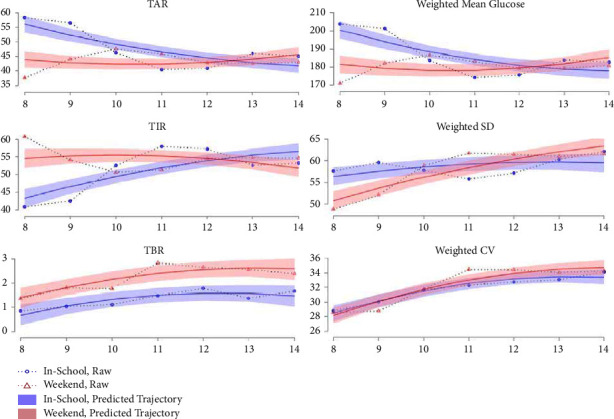
Model-derived mean trajectories with 95% confidence intervals are displayed for each CGM metric by setting (in-school vs. weekend) with raw means overlaid. TAR: time above range; TIR: time in range; TBR: time below range; SD: standard deviation; CV: coefficient of variation.

Weekend CGM metrics were clinically similar to in-school for school hours (Table 3). In regression models for school hours, weekends, compared to in-school, were on average associated with significantly higher TIR (54.5 ± 24.6% vs. 51.0 ± 23.4%, *p* < 0.001) and significantly lower TAR (43.4 ± 25.5% vs. 47.6 ± 24.2%, *p* < 0.001), mean glucose (180.3 ± 44.4 mg/dL vs. 186.5 ± 41.0 mg/dL, *p* < 0.001), and SD (58.0 ± 21.6 vs. 58.7 ± 16.9, *p* < 0.001) (Table 6) during school hours. On average, there was no significant difference in TBR (*p* = 0.99) or glucose CV (*p* = 0.06) between in-school and weekend school hours. There was a significant interaction between hour and setting (in-school vs. weekend) when investigating TAR, TIR, weighted mean glucose, weighted SD (all *p* < 0.001), and weighted CV (*p* = 0.022), suggesting that the hourly changes noted in these metrics are significantly different than those on the weekend. Model-derived predictions overlayed with raw data for each CGM metric in this comparison are included in Figure 1.

### 3.3. Comparison of Virtual School CGM Metrics

**Figure 2:**
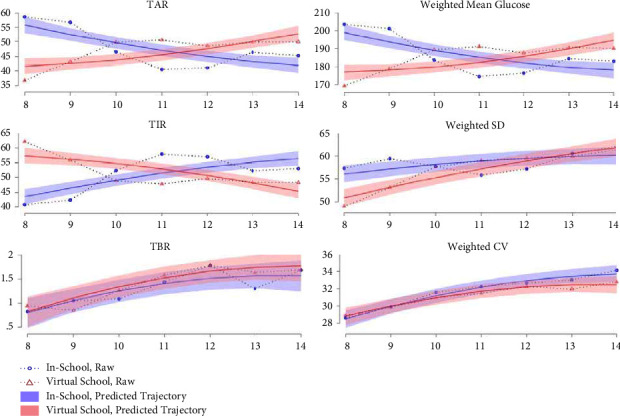
Model-derived mean trajectories with 95% confidence intervals are displayed for each CGM metric by setting (in-school vs. virtual school) with raw means overlaid. TAR: time above range; TIR: time in range; TBR: time below range; SD: standard deviation; CV: coefficient of variation.

**Figure 3:**
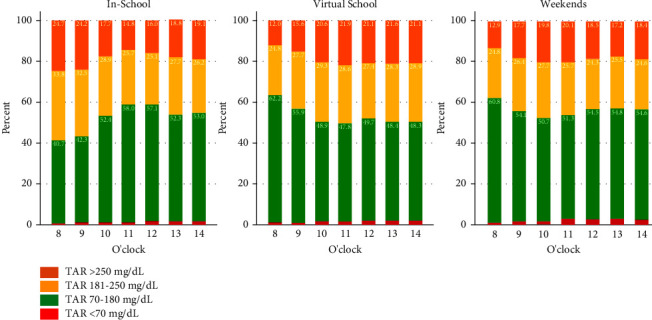
Stacked bar graphs of hourly measures time in range (TIR), time above range (TAR), and time below range (TBR) for in-school, virtual school, and weekend times. TAR is sub-categorized by level 1 (glucoses 181–250 mg/dL) and level 2 (glucoses >250 mg/dL). TIR is noted to increase, and TAR decrease, over the first half of the school day, a pattern not duplicated with virtual school or weekends.

As with weekends, virtual school CGM metrics were clinically similar to in-school during school hours (Table 4). In regression models for school hours, virtual school, compared to in-school, was associated with significantly higher TIR (*p* < 0.001) and significantly lower TAR (*p* < 0.001), mean glucose (*p* < 0.001), and glucose SD (*p* < 0.001) on average during school hours (Table 6). There was no significant difference in TBR (*p* = 0.69) and glucose CV (*p* = 0.054) between virtual school and in-school hours on average. As with weekend values, there was a significant interaction between hour and virtual school for TAR, TIR, weighted mean glucose, weighted SD (all *p* < 0.001), and weighted CV (*p* = 0.018). Model-derived predictions overlayed with raw data for each CGM metric in this comparison are included in Figure 2. The pattern of TIR, TAR, and TBR during virtual school more closely resembled that of the weekend, rather than in-school (Figure 3).

## 4. Discussion

In this observational analysis, we identified several interesting findings in CGM metrics during school hours. Contrary to our hypothesis, the CGM metrics during school hours were statistically better on weekends and in virtual school, though clinically they appeared similar. However, many CGM metrics improved during in-person school hours, a trend which was not replicated on weekends or during virtual school in the same time window. The slightly better CGM metrics on weekends and in virtual school between 8 AM and 3 PM were mostly driven by differences in the early morning hours (between 8 and 10 AM), the same time when children in-school are attaining significant improvements in TIR. These findings signal that the in-person school day may have unique implications for glycemia.

Children in this cohort were beginning in-person school with substantially higher TAR compared to similar times on the weekends, particularly with blood glucoses over 250 mg/dL. Though this may be related to wake times, we did not see as great a rise in TAR later in the morning for weekends or virtual school. Other child or family factors may play a role, including early morning eating habits, activities, school or home-related stressors, or insulin dosing decisions to prevent hypoglycemia on the way to school, as parental fear of hypoglycemia may lead to practices to keep blood glucoses higher [14].

The subsequent fall in TAR and rise in TIR during in-person school hours by nearly 20 percentage points was a strong pattern that persisted when adjusting for age, duration of diabetes, hemoglobin A1c, and insulin pump use. This finding was reflected by the improvement in mean glucose during in-person school hours. However, the relationship between setting and measures of glycemic variability from this analysis is limited. Though there was a significant interaction between setting and time of day for SD, the differences in SD observed between settings were not clinically significant, and we did not find significant differences in CV, which tended to increase regardless of setting over school hours. Other measures of glycemic variability may be explored in future research to better describe the amplitude, direction, and timing of excursions in school [15].

To account for the changes seen in TIR, mean glucose, and TAR, we speculate that some aspect(s) of the in-person school day may help to optimize glycemia. One such variable may be the predictable routines created by the school day with structured classroom activities, physical activity, and mealtimes, which limit opportunities to snack between meals. Regular routines are predictors of diabetes self-management practices [16] and treatment adherence [17]. Interestingly, the CGM data during virtual school hours more closely resembled weekend data, suggesting that school day routines may be disrupted. We have previously shown that parents perceived a decline in some diabetes self-management habits when their children transitioned to virtual learning due to the COVID-19 pandemic, particularly with regard to physical activity and snacking between meals [18]. Given the retrospective nature of this analysis, we were unable to assess how routines differed across in-person school, weekends, and virtual school and the relationship with TIR and other metrics.

For our sampling window of school hours (8 AM to 3 PM), the small proportion of children (5%) in our study meeting a TIR of 70% was striking. In pediatric cohorts without an automated insulin delivery (AID) system, mean TIR has been reported to be 40–61%, with values differing by country and by use of multiple daily injections or insulin pumps [19–21]. In one real-world European cohort, 14% of children using real-time CGM with multiple daily injections and 28% of children using an insulin pump with real-time CGM achieved a TIR of 70%, though they did not impose any time-period restrictions and their sample included all youth less than 18 years of age [21]. Historically, European children tend to achieve hemoglobin A1c targets more often than children in the United States [22]. To date, there is some question about whether this TIR goal is realistic in pediatric populations, as using CGM may not on its own lead to higher TIR in children [23]. This may change with the increasing use of AID systems, as children are increasingly able to attain a mean TIR close to 70% in clinical trials [24–27].

The differences noted during school hours were largely not seen when comparing 24-hour data across these settings. This is not unexpected as the 24-hour data include a larger period of time (e.g., overnight) when the children are in other environments and additional factors are contributing to glycemia. The signal of the change in glycemia during school hours specifically suggests a potential benefit to intervening during this window for glycemia and other possible implications, such as for social interactions, mood, and learning.

Insulin pump use, along with age, duration of diabetes, and hemoglobin A1c were all clinical predictors of TIR in school. These findings are consistent with expected predictors for TIR generally. Hemoglobin A1c is associated with TIR [28], and younger age and shorter duration correlate with lower hemoglobin A1c [8]. We did not observe differences in TIR by race and ethnicity or socioeconomic status, both of which have been shown to have associations with glycemic outcomes, though variably [19, 29]. This may be related to the relative homogeneity of our sample, as the demographics of our sample differ somewhat from our clinical population (13% coming from racial and ethnic minoritized groups, ∼40% with public insurance). We reviewed the characteristics of subjects excluded for missing >1 week of data (*n* = 84) and data sharing turned off (*n* = 47). They were similar to our cohort in terms of race and ethnicity (95% non-Hispanic white), but a greater proportion relied on public insurance exclusively at 49%. This reflects well-described inequities in access to diabetes devices [30, 31], including barriers with insurance coverage leading to interrupted CGM use [32, 33].

Strengths of this study include the large cohort and assessment of hourly data over a 4-week period with two comparison periods. This study does have limitations. The homogenous study population likely reflects inequities in technology access, which affects the generalizability of our findings to a more diverse population of children with type 1 diabetes. Our comparisons should also be interpreted with caution. Sleep habits, eating patterns, and physical activity (e.g., sports participation) may differ substantially for some children on the weekend.

Additionally, the virtual school data were obtained during the COVID-19 pandemic when families were likely experiencing many stressors that may affect glycemic control, including concerns for the risks of illness in people with diabetes, economic hardships, and the complexities of engaging in virtual school for the first time [18]. Though some forms of virtual school predated the pandemic, COVID-19 introduced the unique situation of parents needing to be engaged in helping to teach their children and manage their children's diabetes during school while simultaneously continuing to work. Furthermore, it is unknown whether these children may have been participating in synchronous or asynchronous classroom activities. All of these factors may affect glycemia and the ability to maintain school day routines.

Regardless, our findings suggest that the in-person school day may affect glycemia, which has implications for clinical practice and research. For children with significant hyperglycemia at the start of school, healthcare providers may seek to understand the contributing factors of patients and their families and provide counseling to address any of these issues. Providers can also emphasize the benefit of school day routines and closer partnership with the school nurse for diabetes management. Future research should prospectively assess CGM data in a broader sample, as correlating daytime routines and management behaviors with CGM data will help determine target modifiable activities for interventions. Optimizing management during the school day through future intervention research may be effective to improve glycemia.

## Figures and Tables

**Table 1:** Background characteristics of the study cohort (*n* = 217).

Characteristic	*N* (%) or mean ± SD
Race/Ethnicity	
Non-hispanic white	208 (96)
Non-white	9 (4)
Sex	
Male	99 (46)
Female	118 (54)
Age (years)	10.5 ± 1.5
Diabetes duration (years)	3.5 ± 2.5
Insulin regimen	
Multiple daily injections	78 (36)
Insulin pump	139 (64)
Insulin dose (units/Kg)	0.8 ± 0.2
Hemoglobin A1c (%)	7.5 ± 0.8 (58.8 ± 8.7 mmol/mol)
A1c ≤ 7% (53 mmol/mol)	62 (29)
A1c > 7% (53 mmol/mol)	155 (71)
BMI z-score	0.4 ± 0.9
Diabetes visits	
<3 per year	24 (11)
3+ per year	191 (89)
Insurance status	
Private (primary)	170 (78)
Public only	47 (22)
Median household income, ±1 K	61.9 ± 18.4
ADI national percentile	59.1 ± 21.5

**Table 2:** Overall (8:00–15:00) and hourly CGM metrics for in-person school.

Metric	Overall	Hourly						
	08:00–15:00	08:00	09:00	10:00	11:00	12:00	13:00	14:00
TAR (%)	47.6 ± 24.2	58.2 ± 24.3	56.5 ± 23.5	46.3 ± 23.1	40.5 ± 23.5	40.9 ± 24.0	46.0 ± 22.7	45.1 ± 22.2
Level 2	19.3 ± 18.2	24.8 ± 21.3	24.4 ± 20.5	17.7 ± 17.4	14.7 ± 15.4	15.8 ± 16.4	18.7 ± 16.6	19.0 ± 16.5
Level 1	28.3 ± 13.7	33.4 ± 14.1	32.1 ± 14.3	28.6 ± 13.8	25.8 ± 13.7	25.1 ± 12.8	27.3 ± 12.4	26.0 ± 12.6
TIR (%)	51.0 ± 23.4	40.9 ± 23.6	42.5 ± 22.7	52.6 ± 22.7	58.0 ± 22.7	57.3 ± 23.0	52.6 ± 21.9	53.3 ± 21.3
TBR (%)	1.3 ± 2.5	0.9 ± 2.3	1.0 ± 2.6	1.1 ± 1.9	1.5 ± 2.9	1.8 ± 2.8	1.4 ± 2.3	1.7 ± 2.6
Level 1	1.1 ± 2.1	0.6 ± 1.5	0.9 ± 2.1	1.0 ± 1.7	1.3 ± 2.6	1.5 ± 2.3	1.2 ± 2.0	1.4 ± 2.2
Level 2	0.2 ± 0.7	0.2 ± 1.0	0.2 ± 0.8	0.1 ± 0.4	0.2 ± 0.6	0.3 ± 0.9	0.2 ± 0.6	0.3 ± 0.7
Mean glucose^†^ (mg/dL)	186.4 ± 41.0	203.7 ± 42.7	201.3 ± 43.1	183.6 ± 38.4	174.3 ± 37.6	175.8 ± 39.4	183.7 ± 38.2	182.7 ± 37.4
SD^†^ (mg/dL)	58.7 ± 16.9	57.7 ± 15.8	59.6 ± 16.2	57.9 ± 17.9	55.8 ± 17.1	57.2 ± 16.2	60.3 ± 16.0	62.2 ± 18.1
CV^†^	31.8 ± 8.0	28.8 ± 7.5	30.0 ± 7.5	31.7 ± 8.1	32.3 ± 8.5	32.7 ± 7.3	33.1 ± 7.6	34.1 ± 8.1

Data displayed as mean ± SD. Abbreviations: TAR, time above range (level 1 181–250 mg/dL, level 2 > 250 mg/dL); TIR, time in range (70–180 mg/dL); TBR, time below range (level 1 : 54–69 mg/dL, level 2 < 54 mg/dL); SD, standard deviation; CV, coefficient of variation. ^†^Values is weighted for the number of readings/hour.

**Table 3:** Overall (8:00–15:00) and hourly CGM metrics for weekend.

	Overall	Hourly						
	08:00–15:00	08:00	09:00	10:00	11:00	12:00	13:00	14:00
TAR (%)	43.4 ± 25.5	37.7 ± 26.4	44.1 ± 27.5	47.6 ± 25.2	45.8 ± 24.9	42.9 ± 24.6	42.7 ± 24.2	43.0 ± 24.6
Level 2	17.8 ± 19.6	12.9 ± 17.0	17.7 ± 20.2	19.8 ± 20.0	20.1 ± 20.5	18.5 ± 19.3	17.2 ± 19.9	18.4 ± 19.8
Level 1	25.6 ± 16.3	24.8 ± 18.7	26.4 ± 17.7	27.7 ± 16.8	25.7 ± 16.0	24.3 ± 14.6	25.5 ± 15.2	24.6 ± 14.4
TIR (%)	54.4 ± 24.6	60.8 ± 26.0	54.1 ± 26.5	50.7 ± 24.4	51.3 ± 23.9	54.5 ± 23.5	54.8 ± 23.1	54.6 ± 23.5
TBR (%)	2.2 ± 4.7	1.4 ± 3.9	1.8 ± 5.2	1.8 ± 4.4	2.8 ± 5.1	2.6 ± 4.6	2.6 ± 4.7	2.4 ± 4.4
Level 1	1.7 ± 3.3	1.0 ± 3.0	1.3 ± 3.7	1.3 ± 2.8	2.1 ± 3.4	2.0 ± 3.3	1.9 ± 3.4	1.9 ± 3.3
Level 2	0.5 ± 2.7	0.3 ± 2.5	0.5 ± 2.8	0.5 ± 3.0	0.7 ± 3.2	0.6 ± 2.7	0.6 ± 2.6	0.4 ± 1.8
Mean glucose^†^ (mg/dL)	180.3 ± 44.4	171.0 ± 38.8	182.0 ± 43.3	186.6 ± 43.2	182.9 ± 45.9	179.4 ± 44.5	179.5 ± 46.5	180.8 ± 46.8
SD^†^ (mg/dL)	58.0 ± 21.6	48.9 ± 20.0	52.2 ± 20.6	58.9 ± 21.2	61.7 ± 20.8	61.4 ± 21.0	61.0 ± 21.8	61.6 ± 21.9
CV^†^	32.3 ± 10.2	18.5 ± 10.4	28.7 ± 9.6	31.8 ± 10.1	34.4 ± 11.0	34.4 ± 9.3	34.1 ± 9.3	34.3 ± 9.7

Data displayed as mean ± SD. Abbreviations: TAR, time above range (level 1 181–250 mg/dL, level 2 >250 mg/dL); TIR, time in range (70–180 mg/dL); TBR, time below range (level 1: 54–69 mg/dL, level 2 <54 mg/dL); SD, standard deviation; CV, coefficient of variation. ^†^Values is weighted for the number of readings/hour.

**Table 4:** Overall (8:00–15:00) and hourly CGM metrics for virtual school.

Metric	Overall	Hourly						
	08:00–15:00	08:00	09:00	10:00	11:00	12:00	13:00	14:00
TAR (%)	47.0 ± 25.0	36.8 ± 24.6	43.2 ± 24.7	49.8 ± 24.5	50.6 ± 24.6	48.6 ± 25.1	49.9 ± 24.5	50.0 ± 23.9
Level 2	19.1 ± 19.0	12.0 ± 15.3	15.6 ± 16.7	20.6 ± 19.7	21.9 ± 20.1	21.1 ± 19.4	21.6 ± 19.7	21.1 ± 19.4
Level 1	27.9 ± 14.1	24.1 ± 15.4	27.7 ± 14.2	29.3 ± 13.6	28.6 ± 13.7	27.4 ± 14.3	28.3 ± 13.5	28.9 ± 13.4
TIR (%)	51.6 ± 24.1	62.2 ± 24.2	55.9 ± 24.2	48.9 ± 23.6	47.8 ± 23.5	49.7 ± 23.9	48.4 ± 23.1	48.3 ± 22.7
TBR (%)	1.4 ± 2.9	0.9 ± 2.6	0.8 ± 2.4	1.3 ± 3.3	1.6 ± 2.9	1.8 ± 3.1	1.6 ± 3.0	1.7 ± 2.7
Level 1	1.1 ± 2.4	0.8 ± 2.3	0.7 ± 2.3	1.0 ± 2.6	1.3 ± 2.5	1.4 ± 2.4	1.3 ± 2.5	1.3 ± 2.2
Level 2	0.3 ± 0.8	0.1 ± 0.6	0.1 ± 0.4	0.2 ± 0.9	0.3 ± 0.7	0.4 ± 1.1	0.3 ± 0.9	0.4 ± 0.9
Mean glucose† (mg/dL)	185.2 ± 41.8	169.3 ± 36.5	178.7 ± 37.5	189.3 ± 41.4	191.0 ± 42.9	187.6 ± 43.1	190.5 ± 43.4	190.1 ± 43.0
SD† (mg/dL)	57.1 ± 17.3	48.9 ± 17.3	53.1 ± 16.4	57.6 ± 16.1	59.0 ± 16.7	59.5 ± 16.7	59.9 ± 17.0	61.4 ± 17.6
CV^†^	31.2 ± 8.3	28.9 ± 8.6	29.9 ± 8.2	31.0 ± 8.3	31.5 ± 8.3	32.2 ± 8.0	32.0 ± 7.9	32.8 ± 8.0

Data displayed as mean ± SD. Abbreviations: TAR, time above range (level 1 181–250 mg/dL, level 2 > 250 mg/dL); TIR, time in range (70–180 mg/dL); TBR, time below range (level 1 : 54–69 mg/dL, level 2 < 54 mg/dL); SD, standard deviation; CV, coefficient of variation. ^†^Values is weighted for the number of readings/hour.

**Table 5:** Clinical and sociodemographic predictors of TIR during in-person school.

Predictor	Unadjusted model *β* (95% CI)	Fully adjusted model *β* (95% CI)	Parsimonious model *β* (95% CI)
Time of day			
Hour		27.59 (23.38, 31.80)^*∗∗∗*^	27.59 (23.38, 31.80)^*∗∗∗*^
Hour^2^		−1.15 (−1.34, −0.96)^*∗∗∗*^	−1.15 (−1.34, −0.96)^*∗∗∗*^
Race			
White	(Ref)	(Ref)	—
Non-white	−7.64 (−20.49, 5.21)	−3.16 (−13.41, 7.10)	—
Sex			
Male	(Ref)	(Ref)	—
Female	1.30 (−3.82, 6.41)	1.18 (−2.84, 5.20)	—
Age	−2.83 (−4.52, −1.15)^*∗∗*^	−1.98 (−3.40, −0.57)^*∗∗*^	−1.95 (−3.35, −0.55)^*∗∗*^
Diabetes duration, years	−1.79 (−2.76, −0.82)^*∗∗∗*^	−0.64 (−1.51, 0.23)	−0.73 (−1.59, 0.12)
Insulin regimen			
MDI	(Ref)	(Ref)	(Ref)
Insulin pump	1.08 (−4.34, 6.50)	5.06 (0.56, 9.55)^*∗*^	5.36 (0.96, 9.76)^*∗*^
Insulin dose (units/Kg)	−27.15 (−37.13, −17.16)^*∗∗∗*^	−7.36 (−16.96, 2.24)	−6.92 (−16.37, 2.52)
Hemoglobin A1c (%)	−12.62 (−15.06, −10.18)^*∗∗∗*^	−11.44 (−13.94, −8.95)^*∗∗∗*^	−11.43 (−13.92, −8.93)^*∗∗∗*^
BMI z-score	−0.33 (−3.36, 2.71)	1.19 (−1.23, 3.60)	—
Insurance status			
Private	(Ref)	(Ref)	—
Public	0.94 (−5.33, 7.22)	0.12 (−4.81, 5.05)	—
ADI national percentile	−0.05 (−0.17, 0.07)	−0.04 (−0.14, 0.05)	—

Notes: For *n* = 195 subjects after excluding subjects who have missing values in any variables. Abbreviations: MDI, multiple daily injections; ADI, area deprivation index Unadjusted model is adjusted by hour and hour^2^. Fully adjusted model includes all predictors. Parsimonious model includes only predictors which are significant or *p* < 0.20 in the fully adjusted model ^*∗*^significant at the *p*<0.05 level ^*∗∗*^Significant at the *p* < 0.01 level ^*∗∗∗*^Significant at the *p* < 0.001 level.

**Table 6:** Regression modeling comparing CGM metric by setting.

	Fully-adjusted model *β*(95% CI)	*p*-value
TAR (%)		
In-school	(Ref)	
Weekend	−32.00 (−38.92, −25.07)	<0.001
Virtual school	−47.50 (−54.66, −40.34)	<0.001
TIR (%)		
In-school	(Ref)	
Weekend	31.84 (25.09, 38.60)	<0.001
Virtual school	47.70 (40.77, 54.63)	<0.001
TBR (%)		
In-school	(Ref)	
Weekend	−0.01 (−1.35, 1.32)	0.985
Virtual school	−0.20 (−1.17, 0.77)	0.691
Mean glucose^†^ (mg/dL)		
In-school	(Ref)	
Weekend	−52.60 (−63.79, −41.41)	<0.001
Virtual school	−74.05 (−85.73, −62.36)	<0.001
Standard deviation^†^ (mg/dL)		
In-school	(Ref)	
Weekend	−17.98 (−23.55, −12.42)	<0.001
Virtual school	−14.47 (−19.03, −9.92)	<0.001
CV^†^ (%)		
In-school	(Ref)	
Weekend	−2.83 (−5.79, 0.13)	0.061
Virtual school	2.45 (−0.04, 4.95)	0.054

TAR, time above range; TIR, time in range; TBR, time below range; SD, standard deviation; CV, coefficient of variation. Fully adjusted model includes all the potential sociodemographic and clinical predictors: hour, hour^2^, interaction term between setting and hour, race, sex, age, duration of diabetes, regimen, body mass index, insulin dose (units/kilogram), hemoglobin A1c, primary insurance, and area deprivation index national percentile. ^†^Values are weighted for the number of readings/hour.

## Data Availability

All data generated or analyzed during this study are included in this article. Further inquiries can be directed to the corresponding author.

## Supplementary Materials

Supplementary Materials Supplement 1 depicts the summary statistics of continuous glucose monitoring metrics for 24-hour periods across the three settings (in-school, weekend, and virtual school).

## References

[1] Holmes B. W., Sheetz A., Allison M. (2016). Role of the school nurse in providing school health services. *Pediatrics*.

[2] Faro B., Ingersoll G., Fiore H., Ippolito K. S. (2005). Improving students’ diabetes management through school-based diabetes care. *Journal of Pediatric Health Care*.

[3] Engelke M. K., Guttu M., Warren M. B., Swanson M. (2008). School nurse case management for children with chronic illness: health, academic, and quality of life outcomes. *The Journal of School Nursing*.

[4] Nguyen T. M., Mason K. J., Sanders C. G., Yazdani P., Heptulla R. A. (2008). Targeting blood glucose management in school improves glycemic control in children with poorly controlled type 1 diabetes mellitus. *The Journal of Pediatrics*.

[5] Izquierdo R., Morin P. C., Bratt K. (2009). School-centered telemedicine for children with type 1 diabetes mellitus. *The Journal of Pediatrics*.

[6] Tonyushkina K. N., Cobb V., Lawson G. (2021). Challenges and opportunities of engaging school nurses in a diabetes care team in a culturally diverse lowincome community – a mixed-methods feasibility study. *Health Behav Policy Rev*.

[7] Pansier B., Schulz P. J. (2015). School-based diabetes interventions and their outcomes: a systematic literature review. *Journal of Public Health Research*.

[8] Foster N. C., Beck R. W., Miller K. M. (2019). State of type 1 diabetes management and outcomes from the T1D exchange in 2016-2018. *Diabetes Technology and Therapeutics*.

[9] Yapanis M., James S., Craig M. E., O’Neal D., Ekinci E. I. (2022). Complications of diabetes and metrics of glycemic management derived from continuous glucose monitoring. *Journal of Clinical Endocrinology and Metabolism*.

[10] Battelino T., Danne T., Bergenstal R. M. (2019). Clinical targets for continuous glucose monitoring data interpretation: recommendations from the international consensus on time in range. *Diabetes Care*.

[11] American Diabetes Association Professional Practice Committee (2022). 14. Children and adolescents: standards of medical care in diabetes-2022. *Diabetes Care*.

[12] Kind A. J. H., Buckingham W. R. (2018). Making neighborhood-disadvantage metrics accessible - the neighborhood atlas. *New England Journal of Medicine*.

[13] University of Wisconsin School of Medicine and Public Health (2019). Area deprivation index version 3. https://www.neighborhoodatlas.medicine.wisc.edu/.

[14] Pierce J. S., Kozikowski C., Lee J. M., Wysocki T. (2017). Type 1 diabetes in very young children: a model of parent and child influences on management and outcomes. *Pediatric Diabetes*.

[15] Guilmin-Crepon S., Carel J. C., Schroedt J., Scornet E., Alberti C., Tubiana-Rufi N. (2018). How should we assess glycemic variability in type 1 diabetes? Contribution of principal component analysis for interstitial glucose indices in 142 children. *Diabetes Technology and Therapeutics*.

[16] Fritz H. (2014). The influence of daily routines on engaging in diabetes self-management. *Scandinavian Journal of Occupational Therapy*.

[17] Greening L., Stoppelbein L., Konishi C., Jordan S. S., Moll G. (2006). Child routines and youths’ adherence to treatment for type 1 diabetes. *Journal of Pediatric Psychology*.

[18] March C. A., Siminerio L. M., Muzumdar R. H., Libman I. M. (2021). Implications of the school day on health behaviors for children with type 1 diabetes: a survey of parent perspectives during the COVID-19 pandemic. *The Science of Diabetes Self-Management and Care*.

[19] DiMeglio L. A., Kanapka L. G., DeSalvo D. J. (2020). Time spent outside of target glucose range for young children with type 1 diabetes: a continuous glucose monitor study. *Diabetic Medicine*.

[20] Urakami T., Terada H., Yoshida K. (2022). Comparison of the clinical effects of intermittently scanned and real-time continuous glucose monitoring in children and adolescents with type 1 diabetes: a retrospective cohort study. *Journal of Diabetes Investigation*.

[21] Cherubini V., Bonfanti R., Casertano A. (2020). Time in range in children with type 1 diabetes using treatment strategies based on nonautomated insulin delivery systems in the real world. *Diabetes Technology and Therapeutics*.

[22] DeSalvo D. J., Miller K. M., Hermann J. M. (2018). Continuous glucose monitoring and glycemic control among youth with type 1 diabetes: international comparison from the T1D Exchange and DPV Initiative. *Pediatric Diabetes*.

[23] Laffel L. M., Kanapka L. G., Beck R. W. (2020). Effect of continuous glucose monitoring on glycemic control in adolescents and young adults with type 1 diabetes: a randomized clinical trial. *JAMA*.

[24] Garg S. K., Weinzimer S. A., Tamborlane W. V. (2017). Glucose outcomes with the in-home use of a hybrid closed-loop insulin delivery system in adolescents and adults with type 1 diabetes. *Diabetes Technology and Therapeutics*.

[25] Brown S. A., Kovatchev B. P., Raghinaru D. (2019). Six-month randomized, multicenter trial of closed-loop control in type 1 diabetes. *New England Journal of Medicine*.

[26] Breton M. D., Kanapka L. G., Beck R. W. (2020). A randomized trial of closed-loop control in children with type 1 diabetes. *New England Journal of Medicine*.

[27] Brown S. A., Forlenza G. P., Bode B. W. (2021). Multicenter trial of a tubeless, on-body automated insulin delivery system with customizable glycemic targets in pediatric and adult participants with type 1 diabetes. *Diabetes Care*.

[28] Beck R. W., Bergenstal R. M., Cheng P. (2019). The relationships between time in range, hyperglycemia metrics, and HbA1c. *J Diabetes Sci Technol*.

[29] Sutherland M. W., Ma X., Reboussin B. A. (2020). Socioeconomic position is associated with glycemic control in youth and young adults with type 1 diabetes. *Pediatric Diabetes*.

[30] Lipman T. H., Smith J. A., Patil O., Willi S. M., Hawkes C. P. (2021). Racial disparities in treatment and outcomes of children with type 1 diabetes. *Pediatric Diabetes*.

[31] Addala A., Auzanneau M., Miller K. (2021). A decade of disparities in diabetes technology use and HbA1c in pediatric type 1 diabetes: a transatlantic comparison. *Diabetes Care*.

[32] Sumnik Z., Szypowska A., Iotova V. (2019). Persistent heterogeneity in diabetes technology reimbursement for children with type 1 diabetes: the SWEET perspective. *Pediatric Diabetes*.

[33] Prahalad P., Addala A., Buckingham B. A., Wilson D. M., Maahs D. M. (2018). Sustained continuous glucose monitor use in low-income youth with type 1 diabetes following insurance coverage supports expansion of continuous glucose monitor coverage for all. *Diabetes Technology and Therapeutics*.

