# Supplementary material for: Comparisons of School-Day Glycemia in Different Settings for Children with Type 1 Diabetes Using Continuous Glucose Monitoring
Source: Pediatr Diabetes. 2023 Mar 9;2023:8176606. doi: 10.1155/2023/8176606 (PMC10623999; doi:10.1155/2023/8176606)
Supplement: Supplementary Materials — Supplement 1 depicts the summary statistics of continuous glucose monitoring metrics for 24-hour periods across the three settings (in-school, weekend, and virtual school). [file 8176606.f1.docx]

**Supplement 1**

Table: CGM metric data for 24-hour periods for In-School, Weekend, and Virtual School

| CGM Metric | In-School | Weekend | p-value | Virtual School | p-value |
| --- | --- | --- | --- | --- | --- |
| Sensor Usage (%) | 95.0 ± 8.5 | 94.9 ± 10.7 | 0.89 | 95.6 ± 9.1 | 0.58 |
| Mean Glucose (mg/dL)† | 181.6 ± 26.8 | 184.7 ± 44.8 | 0.22 | 183.5 ± 32.0 | 0.25 |
| Standard Deviation (mg/dL)† | 65.2 ± 13.7 | 63.8 ± 14.4 | 0.009 | 63.1 ± 14.3 | 0.003 |
| Level 2 time above range (%) | 17.9 ± 11.0 | 18.5 ± 12.9 | 0.25 | 18.3 ± 13.4 | 0.40 |
| Level 1 time above range (%) | 30.7 ± 8.2 | 30.5 ±9.4 | 0.69 | 31.3 ± 9.0 | 0.45 |
| Time in range | 48.8 ± 14.9 | 48.2 ± 16.6 | 0.35 | 48.0 ± 16.7 | 0.38 |
| Level 1 time below range (%) | 1.7 ± 1.8 | 2.0 ± 2.1 | 0.01 | 1.6 ± 2.0 | 0.47 |
| Level 2 time below range (%) | 0.3 ± 0.5 | 0.5 ± 1.5 | 0.02 | 0.2 ± 0.6 | 0.78 |

Table notes: Summary statistics (mean ± SD) are presented. The significance testing assesses comparisons between in-school and weekend and in-school and virtual school using Student’s T-test.

†Values is weighted for the number of readings/hour.
